# Monitoring ratio of carbon to nitrogen (C/N) in wheat and barley leaves by using spectral slope features with branch-and-bound algorithm

**DOI:** 10.1038/s41598-018-28351-8

**Published:** 2018-07-03

**Authors:** Xingang Xu, Guijun Yang, Xiaodong Yang, Zhenhai Li, Haikuan Feng, Bo Xu, Xiaoqing Zhao

**Affiliations:** 1 Key Laboratory of Quantitative Remote Sensing in Agriculture of Ministry of Agriculture P. R. China, Beijing Research Center for Information Technology in Agriculture, Beijing, 100097 China; 2 National Engineering Research Center for Information Technology in Agriculture, Beijing, 100097 China

## Abstract

Ratio of carbon to nitrogen concentration (C/N) that can illuminate metabolic status of C and N in crop leaves is one valuable indicator for crop nutrient diagnosis. This study explored the feasibility of using spectral slope features from hyperspectral measurements with Branch-and-Bound (BB) algorithm to monitor leaf C/N in wheat and barley. Experimental data from barley in 2010 and wheat in 2012 were collected and used. The analyses prove that leaf C/N is closely related to leaf N concentration (LNC), which implies that it is feasible to apply spectral technology to monitor leaf C/N in that LNC may have been effectivly estimated by hyperspectral measurements. The results also show that many spectral slope features proposed in this study exhibit the significant correlations with leaf C/N. The best slope feature could evaluate changes of leaf C/N well, with *R*^2^ of 0.63 for wheat, 0.68 for barley and 0.65 for both species combined, respectively. using BB algorithm with input of optiaml four slope features can improve the accuracy of leaf C/N estimations with *R*^2^ over 0.81. It is concluded that using the spectral slope new features with BB method appears very promising and potential for remotely monitoring leaf C/N in crops.

## Introduction

The metabolic status of carbon (C) and nitrogen (N) as two essential elements of crop plants has significant influence on the ultimate formation of yield and quality in crop production^1–4^. Leaf is the major organ of plant photosynthesis and physiological activity, and in leaf tissues the ratio of carbon to nitrogen (C/N), defined as the ratio of LCC (leaf carbon concentration) to LNC (leaf nitrogen concentration), can help people to understand and quantify the carbon and nitrogen metabolism in crop plants, and is a good indicator for synthetically diagnosing the balance of carbon and nitrogen, nutrient status, growth vigor and disease resistance in crops^5^. Thus, it is very significant for dynamic regulation in crop fields to monitor changes of leaf C/N quickly and accurately and in real time.

The traditional methods of determinating crop C or N status rely substantially on sampling from fields and analysis in laboratories, but have the disadvantages of either destructive measurements with too much energy and time cost or hysteretic evaluations because of chemical assay in labs^6^. Currently, some non-destructive ways have been presented for the evaluation of crop matter status including chlorophyll meters such as SPAD-502 and leaf colour charts^7–9^. Yet, those methods are only applicable to small areas due to the requirements of contacting with leaves, and meanwhile, pay close attention to individual leaves but a little difficult for population status of crop plants, especially in larger regions^10,11^. In contrast, remote sensing of canopy reflectance acts an essential role in detecting physiological parameters of crop in field, with rapid and large-spatial-area measurement capabilities. Especially, hyperspectral remote sensing with hundreds of narrow bands can probe into the subtle changes of biochemical components (such as leaf chlorophyll content, nitrogen status and water stress) in crop, and markedly characterizes the non-destruction and quickness. Analyzing spectral characteristics and developing newly effective spectral variables to monitor biochemical parameters in crops have become the focused interest in the evaluation of crop growth^11–17^.

Nowadays, the mechanism and methods of estimating leaf C/N with remote sensing techniques, especially ground-based hyperspectral measurements, are still placed in the exploring stage, and there are only a few studies to be carried out. For example, Shi *et al*.^18,19^ probed into the feasibility of estimating C/N in dried leaves of vegetation with the preprocessed hyperspectral data, and analyzed the relationships between spectral absorption features of bands centered at 2100 nm and the different levels of LNC and LCC, and that was a beneficial try of quantitative remote sensing for leaf C/N. Xue *et al*.^20^ suggested that NDVI (normalized difference vegetation index) based on the combination of the two spectral bands at 710 and 1650 nm from multi-spectral reflectance of rice canopy could estimate leaf C/N at late growth stage of rice. Feng *et al*.^21^ established the quantitative relationships between leaf soluble sugar to nitrogen ratio in winter wheat and spectral parameters. Zhou *et al*.^22^ found that the normalized absorption depth of 672 nm could monitor leaf C/N in rice. These studies prove that it is feasible to detect leaf C/N in crop plants with spectra reflection, especially using hyperspectral data. However, in current researches, there are usually only a few sensitive bands of reflection to be applied for assessing leaf C/N, and as far as hyperspectral data with hundreds of narrow spectral bands are concerned, it seems that an enormous number of the unused bands in hyperspectral data are redundant or a little waste of data. Beyond that, the quantitative models that employ the sensitive bands or their derived variables characterizing crop growth status may appear instable when extrapolated to other sites and years, and this is probably because a few sensitive bands that vary in space and time due to influences of soil background, view-sun-target geometry, atmospheric state, vegetation canopy structure or properties, may become insensitive to the corresponding physiological parameters of crop plants^23–28^. On the other hand, high correlations between the neighbouring bands of hyperspectral reflectance data can also result in the phenomena that the sensitive bands as previously determined may become insensitive now, by contrast the neighbouring bands seem effective. Therefore, it is still necessary to conduct further research on how to mine the potential information of a good deal of bands from hyperspectral measurements to evaluate crop parameters such as leaf C/N for weakening some disadvantages due to using only a few wavelengths.

From some current studies on hyperspectral monitoring of vegetation physiological parameters such as N status, chlorophyll and so on, it can be seen that the shapes of the peaks or valleys in hyperspectral reflectance curves display the discrepancies because of the differences in amounts or levels of biophysical or biochemical parameters in vegetation^15,29–32^. Especially, variations of N as the most demanding nutrient element for crop developments often lead to the change of amounts or levels not only for biochemical constitutes closely related to N such as chlorophyll, but also for other ingredients involved with C such as soluble sugar due to the interactions between N and C metabolism^33,34^, which may be well reflected indirectly by the tall, low, broad or narrow shape discrepancies of reflectance curves of crop canopy spectra, and maybe these discrepancies can be also used to evaluate C/N in crop leaves. Xu *et al*. proposed new spectral variables, slope features from canopy reflectance curves to well estimate LNC in barley based on the differences of spectral curves^35^. The information from slope features designed to describe the discrepancies of peaks or valleys of a spectral curve with dozens of wavelengths should be more abundant and meaningful than the other spectral indices using only a few sensitive wavelengths, and maybe helps to evaluate leaf C/N effectively. On the other hand, it should also be more rational to apply multiple wavebands to assess the ratio of carbon to nitrogen since the ratio of C/N is related with two elements of C and N for crop development, but not one. Hence, this present study intended to explore the spectral slope features to monitor leaf C/N for guiding field management and fertilizer strategy. Furthermore, a new method, Branch-and-Bound (BB) algorithm that may find the best subset of variables without examining all possible subsets when selecting numbers of variables in a multiple regression analysis was coupled with the slope features for accuracy improvement of estimating leaf C/N.

The objectives of this study were (1) to assess the analysis capability of hyperspectral reflectance data for monitoring leaf C/N in wheat and barley; (2) to evaluate the performance of spectral slope features proposed for effectiveness of estimating leaf C/N in two crops, and (3) to use BB algorithm with slope features to improve the accuracy of leaf C/N estimates.

## Data and Methods

### Study areas and data

#### Study sites

The study area for barley is situated in Hailar Nongken, China’s Inner Mongolia Autonomous Region, where barley is used primarily as malting barley for beer production, and the region has a large planting area with about one-third of the total malt-barley areas in China. In this present work, the typical 38 barley fields with the regular management of fertilizer and irrigation were selected to collect the experimental data. In the 38 fields, two major cultivars of barley, Kenpi 7 (31 fields) and Ganpi 4 (7 fields) were sown during the date range from May 15th to 30th, 2010. Data acquisition in fields for barley was conducted between 8th and 10th July 2010.

The experimental data for wheat were derived from National Experiment Station for Precision Agriculture (40°10.6′N, 116°26.3′E) in Xiaotangshan town, Changping District, Beijing, China. This experiment station has been used for precision agriculture research since 2001. In this study, the experimental data of wheat were collected at 30 plots in 6 fields with each size of 30 m × 10 m (5 plots each field) during the four wheat growth stages, flag-leaf, anthesis, filling, and milk-ripe. The four cultivars of wheat, Nongda 211 (low Grain Protein Content (GPC), 2 fields), Jingdong 8 (medium GPC, 2 fields), Jing9843 (medium GPC) and Zhongmai 175 (high GPC) were sown on September 24th, 2011. For wheat fields there were the normal irrigation and fertilization managements. The data acquisitions of wheat were conducted on April 28th, May 10th, 21st and 31st, 2012, respectively.

#### Data acquisition

Data collections consist of two steps. Firstly, canopy reflectance measurements were performed in fields. For barley, an ASD spectrometer (FieldSpec Pro VNIR, Anaytical Spectral Devices, Inc., USA) with a range from 350 nm to 1050 nm was utilized to measure reflectance data of barley canopies from all of 38 fields in July 2010. When measuring reflectance spectra, the ASD instrument is fitted with a 25° field of views from a height of 1.0 m above barley canopies under sunny conditions. Each spectral measurement in fields was done by averaging 20 times to acquire reliable mean estimates, and a reference panel measurement was taken before and after spectral measurement each time for getting the black and baseline reflectance. For wheat, the canopy spectral reflectance data were measured with an ASD spectrometer (FieldSpec Pro FR, Analytical Spectral Devices, Boulder, Co., USA) with a spectral range of 350–2500 nm and a view angle of 25° in April and May 2012, and the other measuring requirements of wheat canopy reflectance were as the same as barley. For the sake of subsequently analyzing leaf C/N, twenty representative plants of two crops were collected as samples while performing canopy reflectance measurement in each sample plot.

Secondly, the determination of leaf C/N for two crops was conducted indoors. All green leaves were separated from the sampled plants indoors, and then de-enzymed at 105 °C, oven-dried at 80 °C to constant weight for chemical analysis. To determine leaf C/N, both LNC (g 100 g^−1^) and LCC (g 100 g^−1^) from dried leaves were measured by an elemental analyzer (vario MACRO cube, Elementar Analysensysteme GmbH, Germany) that is a macroelement analyzer and applicable to either non-uniform or low element content samples such as soil and vegetation, and able to simultaneously make the determination of nitrogen and carbon in samples. After acquisition of LNC and LCC, leaf C/N was calculated as the ratio of LNC to LCC.

### Methods

#### Preprocessing of hyperspectral data

Considering that measurements of canopy reflectance in some fields encountered cloudy conditions, it is very helpful to use normalized reflectance techniques to suppress the illumination differences and improve the spectral comparability. The following preprocessing of canopy reflectance measurements was carried out in term of the reference methods^36,37^. Firstly, the spectral curves below 400 nm were truncated on account of extreme noise out of the range, the common spectral bands of reflectance data from two ASD instruments, 400–1050 nm were used to analyze the relationship between leaf C/N and spectral variables from two crops, and about 650 bands were thereby adopted in this paper. Then, smoothing the reflectance spectral curves was conducted with a simple average over blocks of five contiguous bands. Finally, the normalization for the smoothed curves was processed by dividing the mean reflectance for that curve. Namely, a spectral curve *ρ*_*i*_ was replaced with *Nr*_*i*_. In this study, all of the subsequent analyses that were related to the slope spectral features utilized the normalized reflectance spectra.

1 $$N{r}_{i}={\rho }_{i}/(\frac{1}{k}\sum _{i=1}^{k}{\rho }_{i})$$

Where *Nr*_*i*_ is the normalized reflectance, *ρ*_*i*_ is the smoothed reflectance before normalization and *k* represents the whole bands of spectral reflectance.

**Figure 1:**
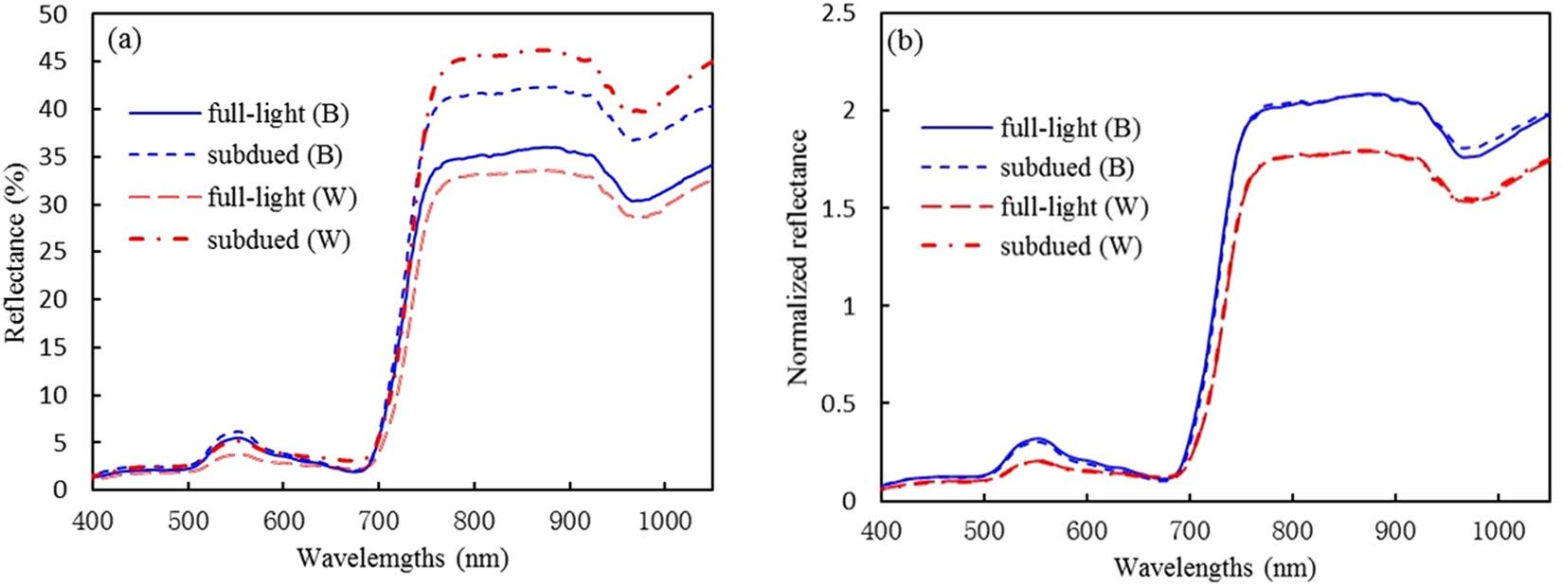
Comparison between canopy reflectance (**a**) and normalized reflectance (**b**) that were derived from barley (B) and wheat (W) samples with the same C/N level and cultivar and measured in full-light and subdued illumination conditions, respectively.

Figure 1a shows that in spite of wheat and barley samples with the same C/N level and cultivar, their corresponding reflectance curves which were measured respectively in full-light and subdued illumination conditions display a significant difference, especially in NIR (near infrared) region. Figure 1b shows the curves after normalization, which reduced effectively the spectral variability due to illumination difference for wheat and barley, respectively.

#### Definition of new spectral features proposed in this study

In a typical crop canopy spectrum, there exist spectral characteristics of the green peak and red well in the visible region due to the strong absorption by pigments (especially chlorophyll) for the blue and red sunlight and the weak reflection for the green. In NIR (near-infrared) region, canopy reflectance rises rapidly because of multiple reflections by microcellular structures in leaf material, and therefore forms a highlighted high reflection platform. C and N are the two essential elements of crop leaves, and their ratio (C/N) can indirectly indicate the proportional changes of specific constituents (such as chlorophyll, enzyme) in leaf tissues. The lower ratio of carbon to nitrogen implies too much N nutrient, which will lead to high chlorophyll content due to close linkage between N and chlorophyll^29,38,39^, and high chlorophyll content will further cause stronger absorption for blue and red light, and thereby results in lower reflectance in visible region, while it will be opposite when there exists higher C/N in leaves. At the same time, the difference of C and N levels in crop will change the internal structure in crop leaves that just influences the reflectance spectra in NIR. Therefore, it is possible that different C/N may be identified by characteristic differences of reflectance spectra.

**Figure 2:**
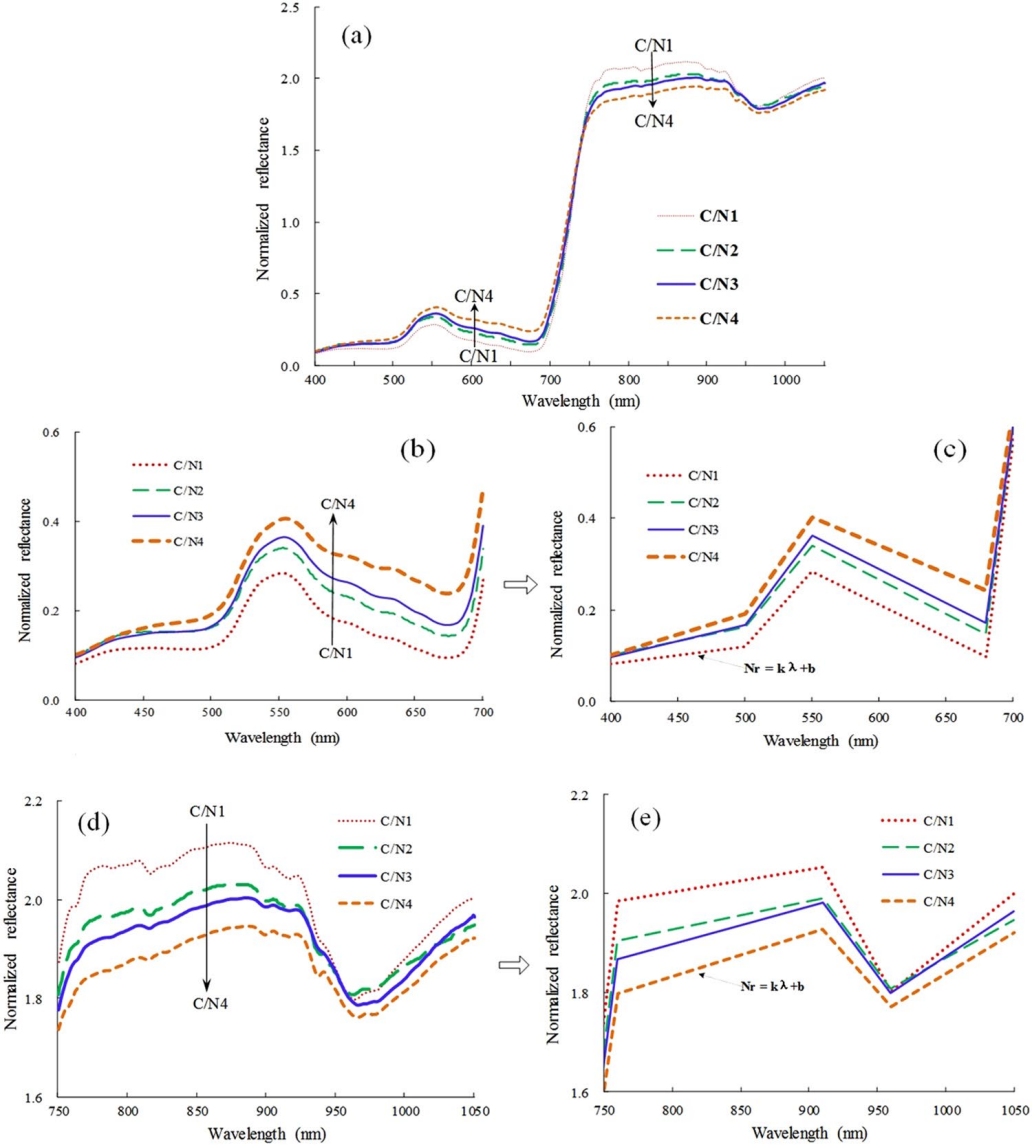
Canopy spectral curves from four different C/N levels ranging from C/N1 to C/N4, 7.5, 9.8, 13.3 and 16.6, respectively. (**a**) Comparison of the whole spectral curves with four C/N levels. (**b**,**c**) The enlarged sketch simply describing the variational features of spectral curves within visible region. (**d**,**e**) The sketch by approximating spectral curve segments into straight line in NIR region.

Figure 2 shows the spectral curves of barley canopies from four C/N levels ranging from C/N1 to C/N4, 7.5, 9.8, 13.3 and 16.6, respectively. It can be observed that canopy reflectance curves under different C/N levels exhibit the tall, low, broad or narrow shape discrepancies. The analyses demonstrate that in the range (500–680 nm) of visible region, canopy reflectance curves with high C/N are taller and broader than ones with low C/N, and the ascent and descent speeds of high C/N curves are also faster than the low C/N ones (Fig. 2b), if both the ascent and descent parts of a spectral curve within this range are approximately looked on as two straight lines, a pair of lines from a higher C/N curve may form a larger inner angle than one pair from a lower C/N curve (see Fig. 2c). In red edge region (680–760 nm), it can be noted that the ascend speed of a reflectance curve with low C/N is slightly quicker than that of high C/N curve, and especially near 740–760 nm, this trend is more obvious (Fig. 2c and e). In NIR region, due to different C/N levels, hyperspectral curves from the ranges of 760–910 nm, 910–960 nm and 960–1050 nm display similar spectral behaviors (Fig. 2d and e). Therefore, it is possible to use the variational shape features of reflectance curves for different spectral regions to monitor dynamic patterns of C/N in crops.

**Figure 3:**
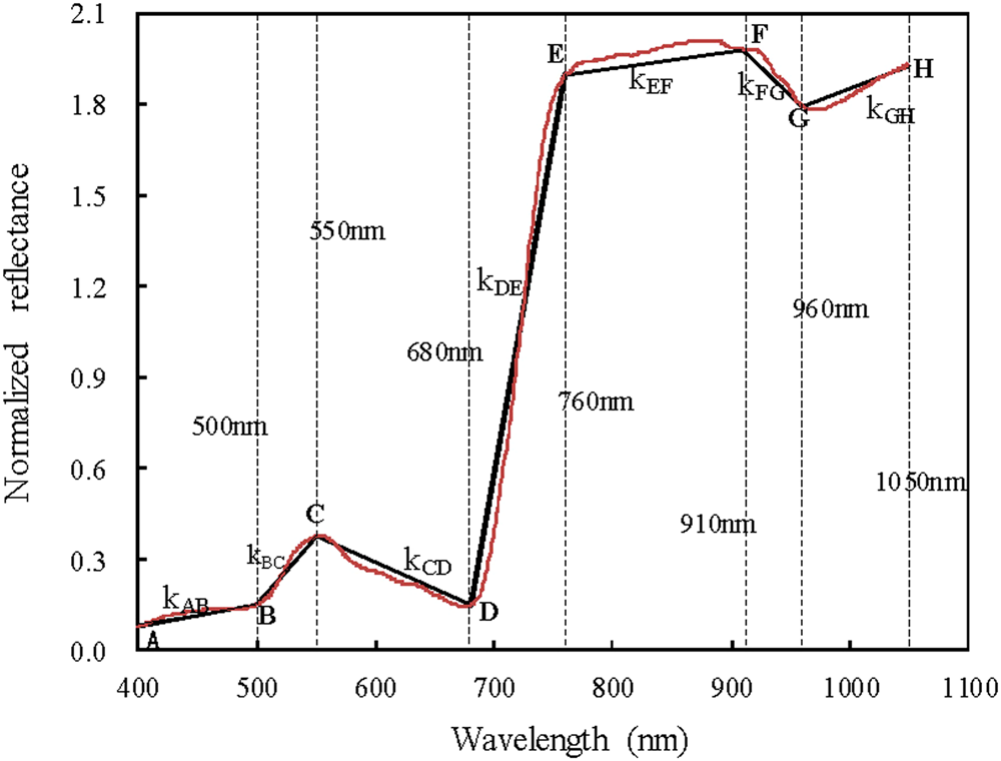
Schematic diagram of slope features extracted from spectral curves.

In view of the above basis, the ascent or descent speeds of spectral curves were proposed as new spectral features to analyze the relationships with leaf C/N in this study. The designs of new spectral features are just as the illustration in Fig. 3. A spectral reflectance curve is divided into many segments such as AB, BC, CD, etc. Each segment is approximately looked on as a straight line, and for each segment the linear fitting is conducted by taking the wavelength as independent variable and the corresponding normalized reflectance as dependent variable, then the slopes of linear fittings (such as *k*_*AB*_, *k*_*BC*_, *k*_*CD*_, etc in Fig. 3) are denoted as the ascent or descent speeds of spectral curves. The equation of calculating slopes is as the following.

2 $$Nr=k\ast \lambda +b$$

Where, *Nr* is normalized reflectance, *λ* wavelength (nm), *k* and *b* are slope and intercept of the fitting equation, respectively.

The determination of spectral curve segments is mainly based on more obvious inflection points between major peaks and valleys in a spectral curve. Here, the wavelengths 500 nm, 550 nm, 680 nm, 760 nm, 910 nm, and 960 nm are determined as the inflection points by averaging 20 typical spectral curves. The definitions of slope spectral features proposed in this present study are as Table 1.

**Table 1:** Definitions of slope features from hyperspectral reflectance curves proposed in this study.

Name	Definition
*K*_*pb*_	Slope within the spectral range of 400–500 nm, namely *k*_*AB*_ in Fig. 3
*K*_*ge*_	Slope *k*_*BC*_ within the green edge (500–550 nm)
*K*_*gprv*_	Slope *k*_*CD*_ within the range from green peak to red valley (550–680 nm)
*K*_*re*_	Slope *k*_*DE*_ within the red edge (680–760 nm)
*K*_*nir*_	Slope *k*_*EF*_ within the range of 760–910 nm
*K*_*nir2*_	Slope *k*_*FG*_ within the range of 910–960 nm
*K*_*nir3*_	Slope *k*_*GH*_ within the range of 960–1050 nm

#### Spectral indices

**Figure 4:**
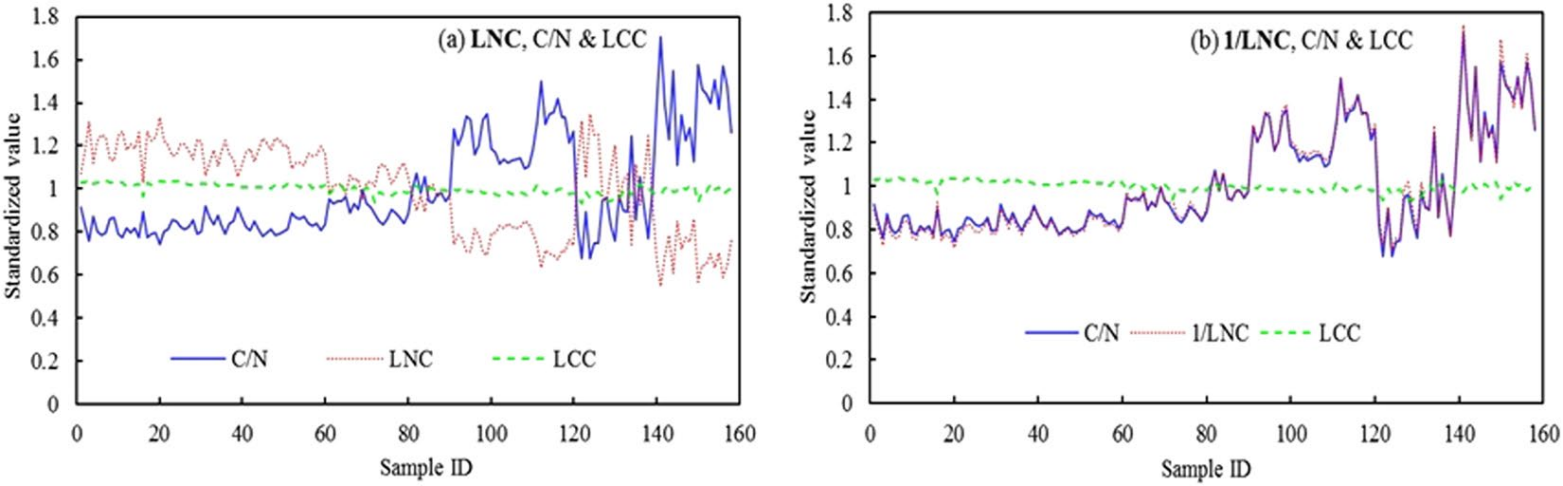
Comparison of change tendencies between C/N, LNC and LCC.

**Figure 5:**
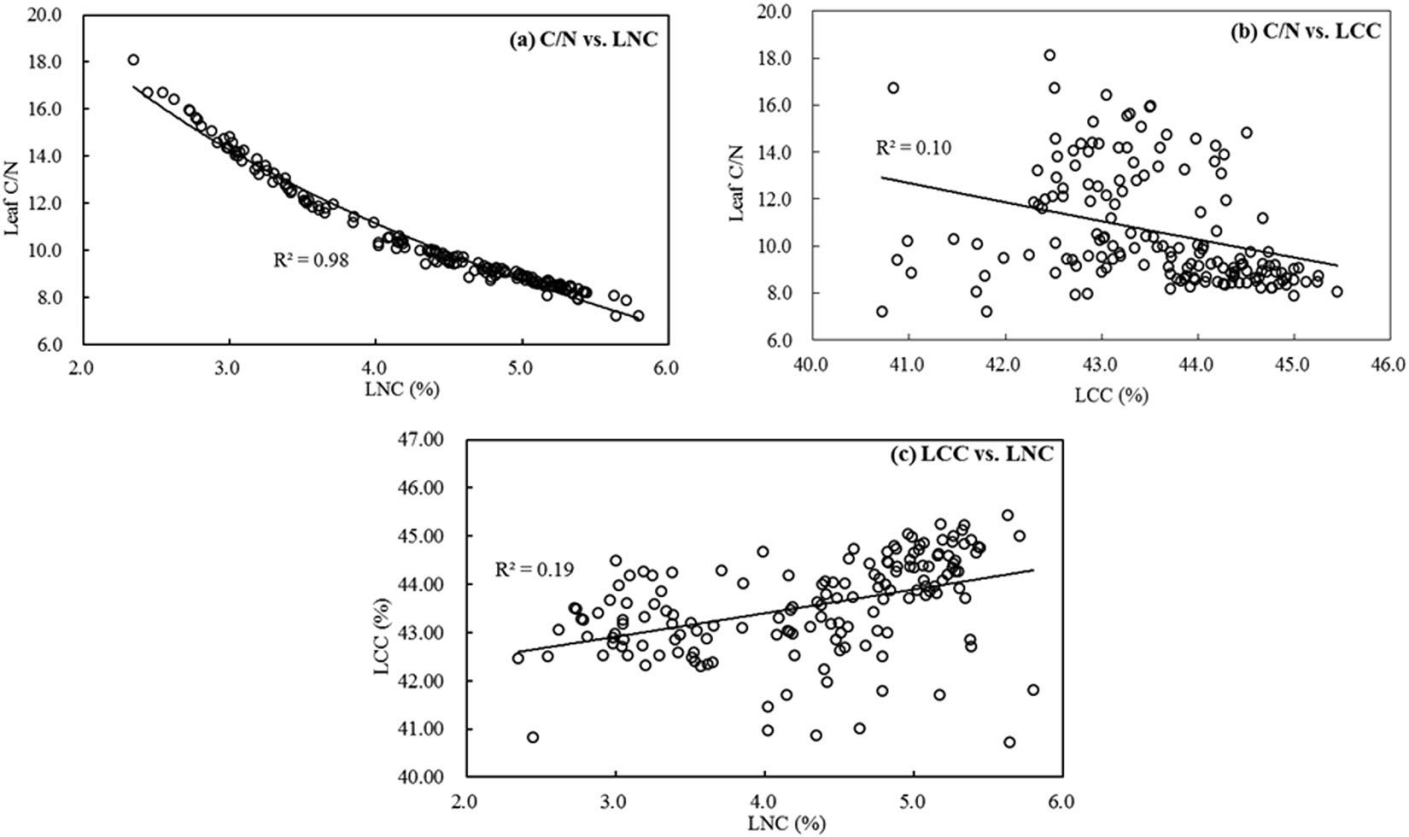
Quantitative relationships between C/N, LNC and LCC.

**Figure 6:**
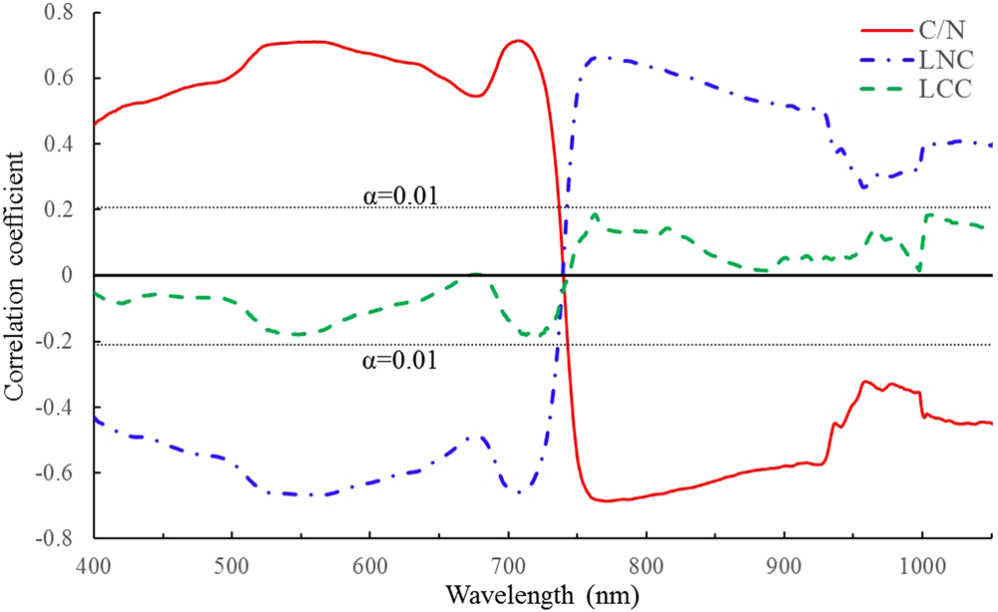
Correlation coefficients between leaf C/N and canopy reflectance.

The analysis results from Figs 4, 5 and 6 show that C/N is closely correlated with N concentration in barley and wheat, which also agreed with the previous researches on leaf C/N from a few of forest vegetations and two rice cultivars^18,22^, and meanwhile, considering the intimate relationship between N and chlorophyll in green leaves^29,38–40^, some spectral indices considered to be good indicators for evaluating N, chlorophyll as well as leaf C/N, were tested in comparison with the better features developed in the study (see Table 2).

**Table 2:** Summary of spectral indices analyzed in the study.

Indices	Formulas	References
**C/N indices**		
*VOG2*	(*R*_*734*_ − *R*_*747*_)/(*R*_*715*_ + *R*_*726*_)	Vogelmann *et al*.^61^ Feng *et al*.^21^
*GIT* ^*#*^	(*R*_*750*–*800*_)/(*R*_*695*–*740*_) − 1	Gitelson *et al*.^62^ Feng *et al*.^21^
**Nitrogen indices**		
*RVI2* ^*#*^	*R*_*810*_/*R*_*560*_	Xue *et al*.^63^
*REP*-*le*^*#*^	Red edge position based on linear extrapolation method	Cho *et al*.^50^
*VIopt*	(1 + 0.45)(*R*_*800*_^2^ + 1)/(*R*_*670*_ + 0.45)	Reyniers *et al*.^57^
*DCNI*	(*R*_*720*_ − *R*_*700*_)/(*R*_*700*_ − *R*_*670*_)/(*R*_*720*_ − *R*_*670*_ + 0.03)	Chen *et al*.^58^
*RI*_*ldB*_	*R*_*735*_/*R*_*720*_	Gupta *et al*.^64^
*SDr*/*SDb*	Sum of 1st derivative within the red edge(680~780 nm) divided by sum of 1st derivative within the blue edge (490~530 nm))	Gong *et al*.^51^ Wang *et al*.^65^
*Dr*	Maximum of 1st derivative within the red edge (680~780 nm)	Gong *et al*.^51^
*MCARI*/*MTVI2*	MCARI: [(*R*_*700*_ − *R*_*670*_) − 0.2(*R*_*700*_ − *R*_*550*_)](*R*_*700*_/*R*_*670*_) MTVI2: 1.5[1.2(*R*_*800*_ − *R*_*550*_) − 2.5(*R*_*670*_ − *R*_*550*_)]/sqrt[(2*R*_*800*_ + 1)^2^ − (6*R*_*800*_ − 5sqrt(*R*_*670*_)) − 0.5]	Eitel *et al*.^59^
**Chlorophyll indices**		
*MSR705* ^*#*^	(*R*_*750*_/*R*_*705*_ − 1)/sqrt(*R*_*750*_/*R*_*705*_ + 1)	Wu *et al*.^66^
*TCARI*/*OSAVI*	*TCARI*: 3[(*R*_*700*_ − *R*_*670*_) − 0.2(*R*_*700*_ − *R*_*550*_)(*R*_*700*_/*R*_*670*_)] *OSAVI:* 1.16(*R*_*800*_ − *R*_*670*_)/(*R*_*800*_ + *R*_*670*_ + 0.16)	Haboudane *et al*.^60^
*mND705*	(*R*_*750*_ − *R*_*705*_)/(*R*_*750*_ + *R*_*705*_ − 2*R*_*445*_)	Sims & Gamon^13^
*MCARI*	[(*R*_*700*_ − *R*_*670*_) − 0.2(*R*_*700*_ − *R*_*550*_)](*R*_*700*_/*R*_*670*_)	Daughtry *et al*.^38^
*NPCI*	(*R*_*680*_ − *R*_*430*_)/(*R*_*680*_ + *R*_*430*_)	Peñuelas *et al*.^67^
*LCI*	(*R*_*850*_ − *R*_*710*_)/(*R*_*850*_ + *R*_*680*_)	Datt^68^
*R*-*M*	*R*_*750*_/*R*_*720*_ − 1	Gitelson *et al*.^69^ Haboudane *et al*.^70^
*NDVIg*-*b*	(*R*_*573*_ − *R*_*440*_)/(*R*_*573*_ + *R*_*440*_)	Hansen *et al*.^14^
*NDVI*	(*R*_*800*_ − *R*_*670*_)/(*R*_*800*_ + *R*_*670*_)	Rouse *et al*.^71^
*MTCI*	(*R*_*750*_ − *R*_*710*_)/(*R*_*710*_ − *R*_*680*_)	Dash & Curran^72^
*NDRE*	(*R*_*790*_ − *R*_*720*_)/(*R*_*790*_ + *R*_*720*_)	Fitzgerald *et al*.^73^
*WRNI*	(*R*_*735*_ − *R*_*720*_)*R_*900*_/[Min*R*_*930*–980_*(*R*_*735*_ + *R*_*720*_)]	Feng *et al*.^74^

^#^ denotes named by this study; *R*_*i*_ denotes reflectance at band *i* (nanometer).

#### Branch-and-bound method

The Branch-and-Bound (BB) method is one general algorithm of searching optimal solutions for various optimization problems, and usually used as a powerful combinatorial optimization tool for feature subset selection problems. When selecting a feature subset from a large number of features to optimize a criterion value over all subsets, exhaustive enumeration of all the subsets is computationally unacceptable as the number of subsets to be considered increases geometrically with the number of features. BB is very efficient to solve this problem because it utilizes partial/selective enumeration rather than exhaustively complete enumeration by using upper and lower estimated bounds of the quantity being optimized, and it can guarantee that the selected subsets yield the globally best criterion value that satisfies monotonicity^41^. Now BB has become one of the most commonly used tools for solving optimization problems. In this study, BB was tested to optimally select the subsets from many slope features for effectively estimating leaf C/N in crops. There are different processing procedures for BB, this study referenced the method from Furnival and Wilson^42^ that is efficient computationally and operationally.

#### Data analysis

First, the correlations among C/N, LNC, LCC and canopy reflectance were investigated to examine the feasibility of applying spectral information to monitor leaf C/N in two crops. Second, barley data from 2010 and wheat data from 2012 were used to analyze the relationships between new features proposed in this study and leaf C/N, and the regression analysis were performed by examining linear, exponential and logarithmic models, and the model with the highest *R*^2^ (determination coefficient) and lowest RMSE (root mean square error) was viewed as the best for every feature. Then these new features were compared with the selected spectral indices for leaf C/N estimates. Finally, BB algorithm was tested to improve the accuracy of C/N estimation with input of new features. *R*^2^ and RMSE were used to evaluate the fitness between the estimated and observed values. In addition, to assess the sensitivity of slope features (SF) to changes in C/N, a noise equivalent C/N (NE ∆C/N) was calculated as:

3 $$\mathrm{NE}\,{\rm{\Delta }}{\rm{C}}/{\rm{N}}={\rm{RMSE}}({\rm{SF}}\,{\rm{vs}}.\,{\rm{C}}/{\rm{N}})/|d({\rm{SF}})/d({\rm{C}}/{\rm{N}})|$$

where RMSE (SF vs. C/N) is the root mean squared error of the relationship SF vs. C/N and |*d*(SF)/*d*(C/N)| is the absolute value of first derivative of SF with respect to C/N. The noise equivalent can be used for direct comparisons among different features with dynamic ranges^43^.

## Results and Analysis

### Relationships between LNC, LCC and C/N

The variation tends between C/N, LNC and LCC were analyzed with barley and wheat data from field measurements (Fig. 4). In order to eliminate the data dimensional difference, the numerical readings on vertical axis in Fig. 4 were the normalized values that each sample datum of C/N, LNC and LCC was divided by their respective averages. From Fig. 4a, it proves that both LNC and LCC are changeable, but the variation of LCC is relatively gentle while LNC varies intensely, so that C/N as the ratio of LNC to LCC exhibited the prominent characteristic of changing markedly with LNC, the symmetry of both C/N and LNC broken lines (see Fig. 4a) can illustrate this viewpoint well. If comparing C/N with the reciprocal of LNC (1/LNC), the change patterns of both are almost the same (Fig. 4b). These analyses demonstrate that C/N is closely related with LNC and has higher correlation with the latter.

Here, a few of quantitative relationships between C/N, LNC and LCC are listed (Fig. 5). The results indicate that there are high correlations between C/N and LNC whether the linear regression or power function fitting, and either of the determination coefficients (*R*^*2*^) exceeds 0.95 (Fig. 5a). In contrast, C/N shows the poor quantitative relationships with LCC (Fig. 5b), neither the linear nor exponential estimation of C/N by LCC displays the effective and acceptable *R*^*2*^. LNC is also poorly related with LCC (Fig. 5c). The analyses from Fig. 5 further manifest that there is the significant correlation between C/N and LNC. Therefore, since employing reflectance data can effectively perform the prediction and monitoring for plant N status^11,14,29,31,44–46^, it is feasible to apply spectral measurements to monitor leaf C/N.

### Leaf C/N correlation with canopy reflectance

The correlation analyses between leaf C/N from barley and wheat data and the normalized hyperspectral reflectance from 400 nm to 1050 nm demonstrate that with significant confidence level (*α* = 0.01), C/N is positively correlated with the reflectance within the range of 400–730 nm, and negatively against the range of 750–1050 nm (Fig. 6). Especially, the peaks of correlation coefficients appeared within the ranges of both 540–560 nm and 710–720 nm (*r* > 0.7), and the lowest in 760–780 nm (*r* < −0.68). In comparison with the correlation coefficient curve of LNC, it can be noted that the trend distribution of C/N correlation curve is almost opposite to that of LNC. On the other hand, LCC is poorly related to canopy reflectance with correlation coefficients in the range from −0.2 to 0.2. The above analyses indicate again that C/N is closely correlated with LNC, and it is worthy of try to use canopy reflectance to estimate and monitor Leaf C/N for crop field managements.

### Relationships of leaf C/N to spectral slope features from reflectance curves

For the sake of establishing the regression models of monitoring leaf C/N, firstly the correlation analyses between C/N and spectral slope features extracted from reflectance curves were conducted, then more sensitive features to leaf C/N were utilized to set up the hyperspectral models for C/N estimates. The analysis results indicate that most of the seven single slope features (see Table 1) are significantly (*p* *<* 0.01) correlated with leaf C/N for wheat, barley or combined data. Especially, considering the slope *K*_*re*_ that uses spectral range of 680–760 nm including the notable red-edge region^47–49^ and has the most negative correlation with C/N, the ratios of slope features between *K*_*re*_ and the other six slopes were applied to explore the relationships with C/N. The analyses find that among the six ratios of slope features (see Table 3), both *K*_*pb*_/*K*_*re*_ and *K*_*ge*_/*K*_*re*_ show the closer relationships with leaf C/N in two crops with the correlation coefficient (*r*) exceeding 0.6, so some combination features related to the two ratios were further proposed and used to examine their performance of estimating leaf C/N from crop canopies. The results demonstrate that the majority of the combined features have the better correlations with leaf C/N. Especially, the combined slope feature (*K*_*ge*_ + *K*_*nir*_)/(2*K_*re*_) displayed the best correlations with C/N in two crops with *r* over 0.78, which may be because the combined feature included the useful information from green edge (*K*_*ge*_), red edge (*K*_*re*_) and high reflection area (*K*_*nir*_) in NIR, and these spectral areas are closely connected with N and C status^21,50,51^.

**Table 3:** Regression analyses between slope features, spectral indices and leaf C/N.

Slope features	Wheat (n = 120)		Barley (n = 38)		Wheat and barley (n = 158)		spectral indices	Wheat (n = 120)		Barley (n = 38)		Wheat and barley (n = 158)	
	*R* ^2^	RMSE	*R* ^2^	RMSE	*R* ^2^	RMSE		*R* ^2^	RMSE	*R* ^2^	RMSE	*R* ^2^	RMSE
*K*_*pb*_	0.40	1.51	0.69	1.73	—	—	*VOG2*	0.33	1.60	0.66	1.84	0.49	1.75
*K*_ge_	0.31	1.62	—	—	—	—	*GIT*	0.32	1.60	0.62	1.93	0.49	1.73
*K*_gprv_	—	—	—	—	—	—	*RVI2*	0.29	1.63	0.57	2.04	0.35	1.94
*K*_*re*_	0.39	1.52	0.54	2.12	0.29	2.04	*REP*-*le*	0.47	1.42	0.55	2.10	0.44	1.81
*K*_*nir*_	0.50	1.38	0.52	2.16	—	—	*VIopt*	—	—	—	—	—	—
*K*_*nir2*_	—	—	0.45	2.33	—	—	*DCNI*	—	—	—	—	—	—
*K*_*nir3*_	—	—	—	—	—	—	*RI*_*ldB*_	0.44	1.45	0.64	1.88	0.56	1.61
*K*_*pb*_/*K*_*re*_	0.40	1.50	0.66	1.81	0.39	1.89	*SDr*/*SDb*	0.39	1.52	0.61	1.96	0.51	1.68
*K*_*ge*_/*K*_*re*_	0.43	1.47	0.59	2.00	0.54	1.64	*Dr*	0.44	1.45	0.68	1.78	—	—
*K*_*gprv*_/*K*_*re*_	0.51	1.37	—	—	—	—	*MCARI*/*MTVI2*	0.28	1.65	—	—	0.28	2.05
*K*_*nir*_/*K*_*re*_	0.56	1.29	0.52	2.15	0.37	1.93	*MSR705*	0.41	1.49	0.63	1.90	0.52	1.67
*K*_*nir2*_/*K*_*re*_	—	—	—	—	—	—	*TCARI*/*OSAVI*	—	—	—	—	—	—
*K*_*nir3*_/*K*_*re*_	—	—	—	—	—	—	*mND705*	0.42	1.48	0.64	1.88	0.55	1.63
(*K*_*ge*_ + *K*_*gprv*_)/(2*K_*re*_)	0.31	1.62	0.59	2.00	0.46	1.77	*MCARI*	—	—	—	—	—	—
(*K*_*ge*_ + *K*_*nir*_)/(2*K_*re*_)	0.63	1.19	0.68	1.78	0.65	1.43	*NPCI*	—	—	0.70	1.71	—	—
(*K*_*ge*_ + *K*_*nir2*_)/(2*K_*re*_)	—	—	0.51	2.19	0.31	2.03	*LCI*	0.30	1.62	0.58	2.03	0.46	1.78
(*K*_*ge*_ + *K*_*nir3*_)/(2*K_*re*_)	0.43	1.47	0.60	1.96	0.48	1.74	*R*-*M*	0.39	1.52	0.64	1.87	0.54	1.65
(*K*_*pb*_ + *K*_*ge*_)/(2*K_*re*_)	0.44	1.46	0.61	1.95	0.54	1.64	*NDVIg*-*b*	0.27	1.67	—	—	0.30	2.03
(*K*_*pb*_ + *K*_*gprv*_)/(2*K_*re*_)	—	—	—	—	—	—	*NDVI*	0.24	1.69	0.49	2.22	0.33	1.98
(*K*_*pb*_ + *K*_*nir*_)/(2*K_*re*_)	0.61	1.23	0.68	1.78	0.43	1.82	*MTCI*	0.49	1.39	0.65	1.85	0.58	1.57
(*K*_*pb*_ + *K*_*nir2*_)/(2*K_*re*_)	—	—	—	—	—	—	*NDRE*	0.36	1.55	0.58	2.02	0.49	1.73
(*K*_*pb*_ + *K*_*nir3*_)/(2*K_*re*_)	—	—	—	—	—	—	*WRNI*	0.38	1.53	0.59	2.00	0.51	1.69

Linear, exponential and logarithm model were applied to make fit, and the results (*R*^2^ and RMSE) of best fit for each variable were showed in the table.

— means there is no significant correlation (*p* < 0.01).

**Figure 7:**
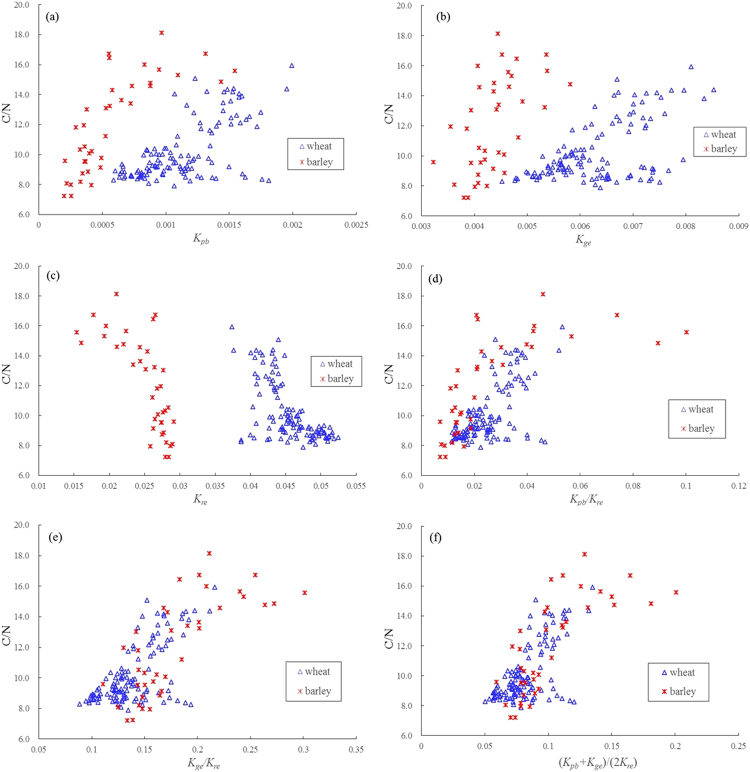
Crop species effects on the correlations between C/N and different types of slope features extracted from wheat and barley data: (**a**) *K*_*pb*_, (**b**) *K*_*ge*_, (**c**) *K*_*re*_, (**d**) *K*_*pb*_/*K*_*re*_, (**e**) *K*_*ge*_/*K*_*re*_, (**f**) (*K*_*pb*_ + *K*_*ge*_)/(2*K_*re*_).

It is also noted from the analysis results that some of single slope features present a closer relationship with leaf C/N for wheat or barley data, but these slope features show weaker or no significant (*P* < 0.01) correlations for the combined dataset. While using simple ratios and combined features, more significant relationships between these features and leaf C/N are observed for the combined dataset. In Fig. 7, the points representing wheat and barley are parallelly grouped but without an overlap for single slope features, *K*_*pb*_, *K*_*ge*_, and *K*_*re*_, it indicates that the single slope features proposed in this study are probably species-dependent as leaf C/N estimators. By contrast, there is a distinct overlapping of wheat and barley for slope ratios *K*_*pb*_/*K*_*re*_ and *K*_*ge*_/*K*_*re*_, and the overlapping trend is further strengthened for the combined feature (*K*_*pb*_ + *K*_*ge*_)/(2*K_*re*_). This demonstrates that the slope ratios and combined features are well correlated to leaf C/N in both wheat and barley, and suitable for evaluating C/N as potential species-independent indicators. At least, they should be appropriate spectral variables for wheat and barley.

### Evaluation of leaf C/N estimates using slope features and spectral indices

Table 3 lists the results of regression analyses between different types of spectral slope features and leaf C/N in wheat and barley. It can be noted from Table 3 that many of slope features exhibit acceptable regression fittings and the combined features present more significant relationships with leaf C/N than single slope features and simple ratios. Especially, the combined feature (*K*_*ge*_ + *K*_*nir*_)/(2*K_*re*_) displayed the stable performance of estimating leaf C/N for wheat, barley or combined dataset with *R*^2^ over 0.6, it had the best relationship with leaf C/N for wheat or combined dataset with a linear function and *R*^2^ values of 0.63 and 0.65 and the corresponding RMSE values to be 1.19 and 1.43, respectively, and also presented a significant relationship only slightly weaker than *K*_*pb*_ for barley with a logarithmic function and *R*^2^ of 0.68 and RMSE to be 1.78 as well as (*K*_*pb*_ + *K*_*nir*_)/(2*K_*re*_). For wheat, the combined feature (*K*_*pb*_ + *K*_*nir*_)/(2*K_*re*_) also showed a better ability to estimate leaf C/N besides (*K*_*ge*_ + *K*_*nir*_)/(2*K_*re*_), with *R*^2^ of 0.61 and RMSE of 1.23. For the combined dataset of both wheat and barley, the other two features *K*_*ge*_/*K*_*re*_ and (*K*_*pb*_ + *K*_*ge*_)/(2*K_*re*_) exhibited a moderate performance as leaf C/N estimators.

**Figure 8:**
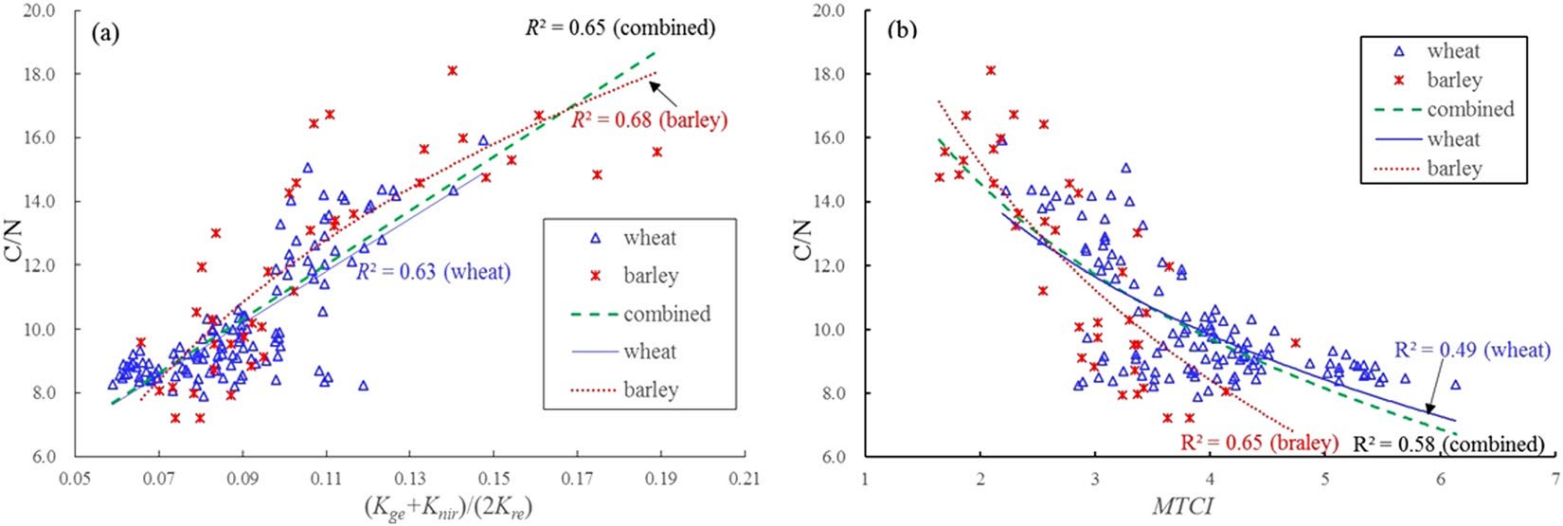
Regression analysis results between leaf C/N and spectral variables derived from wheat and barley data: (**a**) slope feature (*K*_*ge*_ + *K*_*nir*_)/(2*K_*re*_), (**b**) spectral index *MTCI*.

The foregoing analyses (Figs 4–6) prove that leaf C/N is well correlated with N concentration in barley and wheat, and some reports suggested that there existed an intimate relationship between N and chlorophyll^29,38–40^. Hence, 22 spectral indices considered to be good indicators of N, chlorophyll as well as leaf C/N were tested to assess C/N in wheat and barley for comparison with slope features. Table 3 shows the results of regression analyses between spectral indices (see Table 2) and leaf C/N in wheat and barley. Among these indices, the two indices *MTCI* and *RI*_*ldB*_ broadly presented the better relationships with leaf C/N, especially *MTCI* showed the best ability to evaluate leaf C/N for wheat or combined data with a logarithmic function and *R*^2^ values of 0.49 and 0.58 and the corresponding RMSE values to be 1.39 and 1.57, respectively. It also had a better capability of estimating leaf C/N for barley but slightly next to the three indices *NPCI*, *Dr* and *VOG2*. It can be observed in Fig. 8 that the overlapping between points representing wheat and barley is not obvious for *MTCI* with higher values for wheat, and by contrast, there is a clear overlap of wheat and barley for slope feature (*K*_*ge*_ + *K*_*nir*_)/(2*K_*re*_), and the sharp slope of the regression line for (*K*_*ge*_ + *K*_*nir*_)/(2*K_*re*_) implies its higher sensitivity to variations of leaf C/N, which shows that the slope feature (*K*_*ge*_ + *K*_*nir*_)/(2*K_*re*_) has a better ability to evaluate leaf C/N in wheat and barley than *MTCI*.

**Figure 9:**
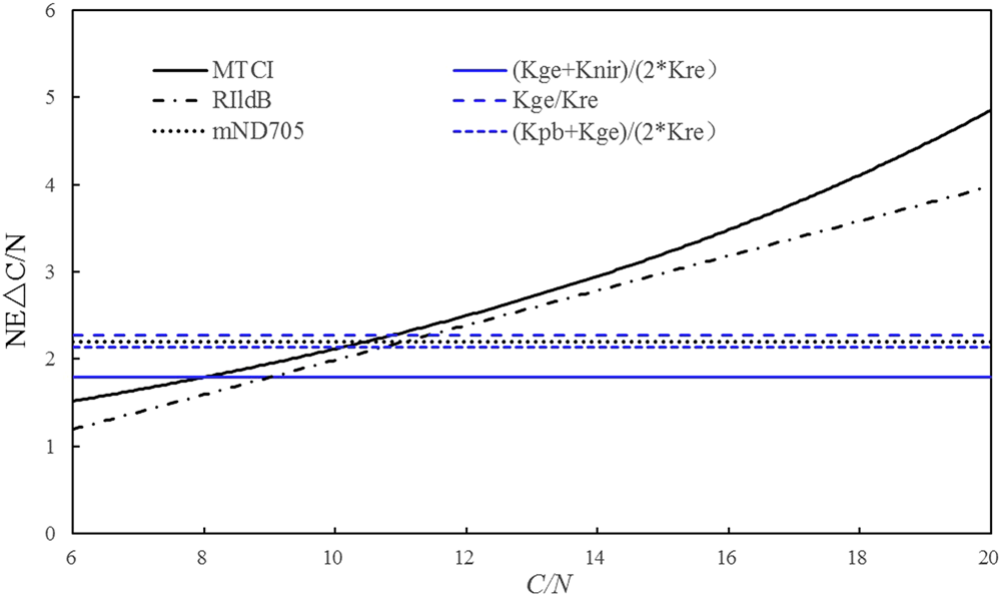
Noise equivalent of ∆C/N for typical slope features and spectral indices evaluated.

Besides, the sensitivity of several typical slope features and spectral indices to C/N changes was evaluated in terms of NE∆C/N. Among 44 of both slope features and spectral indices, the six that performed well for leaf C/N estimates in wheat and barley were selected to compare their sensitivity to leaf C/N changes. Figure 9 shows the sensitivity results of the six variables. It can be observed that the noise equivalents of both *MTCI* and *RI*_*ldB*_ are similar and that of the three *mND705*, *K*_*ge*_/*K*_*re*_ and (*K*_*pb*_ + *K*_*ge*_)/(2*K_*re*_) are closely parallel to each other, but the former two have the smaller NE∆C/N values when C/N is less than 10.8, which indicates that both *MTCI* and *RI*_*ldB*_ are sensitive for C/N < 10.8 by comparison with the latter three. Although the slope feature (*K*_*ge*_ + *K*_*nir*_)/(2*K_*re*_) displays higher NE∆C/N values than that of *MTCI* and *RI*_*ldB*_, for C/N < 8, (*K*_*ge*_ + *K*_*nir*_)/(2*K_*re*_) shows the smallest NE∆C/N values with regards to C/N change when C/N is greater than 8, and its behavior of NE∆C/N is also invariant through the entire range of C/N variation, which demonstrates that (*K*_*ge*_ + *K*_*nir*_)/(2*K_*re*_) is less influenced by saturation caused by C/N changes, and at least it should be appropriate indicator for leaf C/N in wheat and barley.

### Leaf C/N estimation using BB with spectral slope features

**Figure 10:**
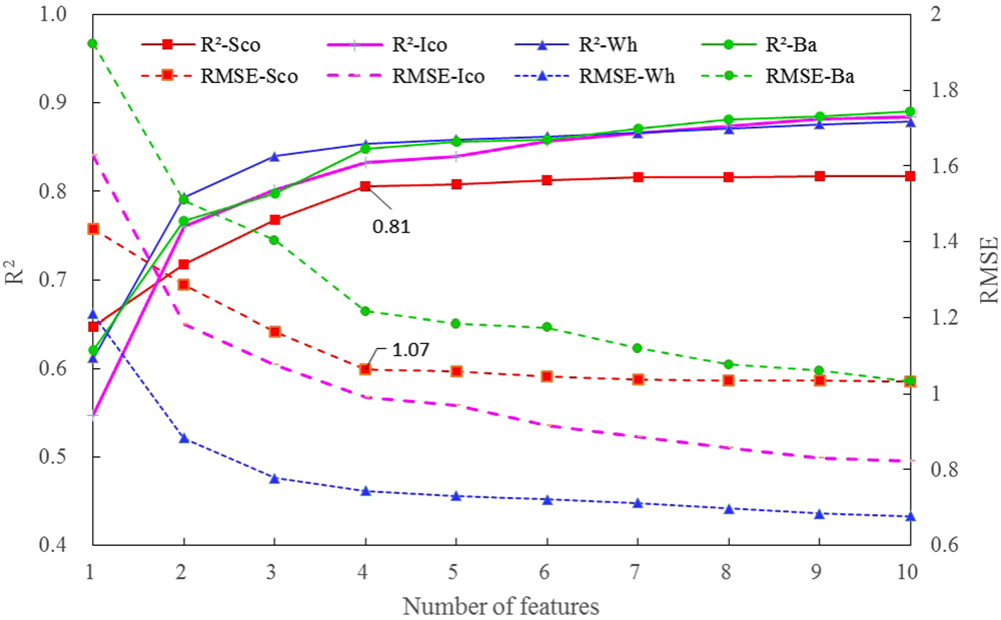
Changes of *R*^2^ and RMSE when using BB to select the optimal subsets with different feature numbers: −Wh means wheat, −Ba barley, and −Sco the combined dataset using slope features respectively, and −Ico denotes the combined dataset for spectral indices.

The Branch-and-Bound (BB) algorithm is an effective tool for selecting optimal subset of features. In this study, BB was tested to obtain the optimal subsets with different feature numbers so as to explore the method of further improving the accuracy of C/N estimation. The proposed 22 features (see Table 3) were input into BB with wheat, barley and combined dataset, respectively, it can be noted that the accuracies of estimating C/N are improved gradually as the feature number of the optimal subset increases (see Fig. 10), and the changes of *R*^2^ and RMSE trend to be stable as the feature number reaches 4, and at this point *R*^2^ arrives at 0.85 for both wheat and barley, 0.81 for combined dataset, but RMSE for wheat is the lowest with the value of 0.74, the worst 1.22 for barley, and the moderate 1.07 for the combined data. In addition, the 22 spectral indices (see Table 2) from the combined dataset were also input into BB in comparison with slope features. The results show that the optimal subset from spectral indices is slightly better than that of slope features when the variable number is 4, with *R*^2^ of 0.83 and RMSE of 0.99, but it seems that *R*^2^ and RMSE from optimal subsets of spectral indices become stable as the number of variables gets to 6.

However, the analysis results also showed that the respective use of wheat, barley or combined data with BB algorithm generated the different optimal feature subsets. Table 4 lists the results of optimal subsets as the feature number is 4, and it can be seen from Table 4 that the regression model of optimal slope feature subset with the combined dataset can effectively estimate leaf C/N in wheat and barley with *R*^2^ values of 0.85 and 0.70 and RMSE values to be 0.76 and 1.71, respectively. When the model of optimal slope subset for wheat data was used to evaluate leaf C/N for barley or combined data comparatively, the estimating accuracy of leaf C/N was obviously reduced in terms of the changes of *R*^2^ or RMSE values. What is worse, the model of optimal subset from barley data could not be used to estimate leaf C/N for wheat and combined data at all. It proves that the optimal feature subsets based on BB algorithm for wheat or barley data are species-dependent for leaf C/N estimates.

**Table 4:** The analyses of C/N estimation using the models of optimal feature subsets from wheat, barley and combined dataset, respectively.

Model names	Optimal feature subset (feature number = 4)	Wheat and barley		Wheat		Warley	
		*R* ^2^	RMSE	*R* ^2^	RMSE	*R* ^2^	RMSE
BB-wh	(*K*_*pb*_ + *K*_*gprv*_)/(2*K_*re*_), (*K*_*pb*_ + *K*_*nir2*_)/(2*K_*re*_), (*K*_*pb*_ + *K*_*nir*_)/(2*K_*re*_), *K*_*nir*_	0.69	1.67	0.85	0.74	—	—
BB-ba	*K*_*pb*_, (*K*_*pb*_ + *K*_*gprv*_)/(2*K_*re*_), *K*_*re*_, *K*_*nir*_	—	—	—	—	0.85	1.22
BB-Sco	(*K*_*ge*_ + *K*_*nir*_)/(2*K_*re*_), (*K*_*ge*_ + *K*_*gprv*_)/(2*K_*re*_), *K*_*nir2*_, *K*_*nir*_	0.81	1.07	0.85	0.76	0.70	1.71
BB-Ico	*MCARI*, *LCI*, *Red*-*edgeNDVI*, *MCARI*/*MTVI2*	0.83	0.99	0.86	0.73	0.76	1.54

— means there is no significant correlation (*p* < 0.01). BB-wh denotes the model of optimal subset output by BB for wheat, BB-ba for barley, and BB-Sco for the combined dataset using slope features respectively, BB-Ico means the model for the combined dataset using spectral indices.

**Figure 11:**
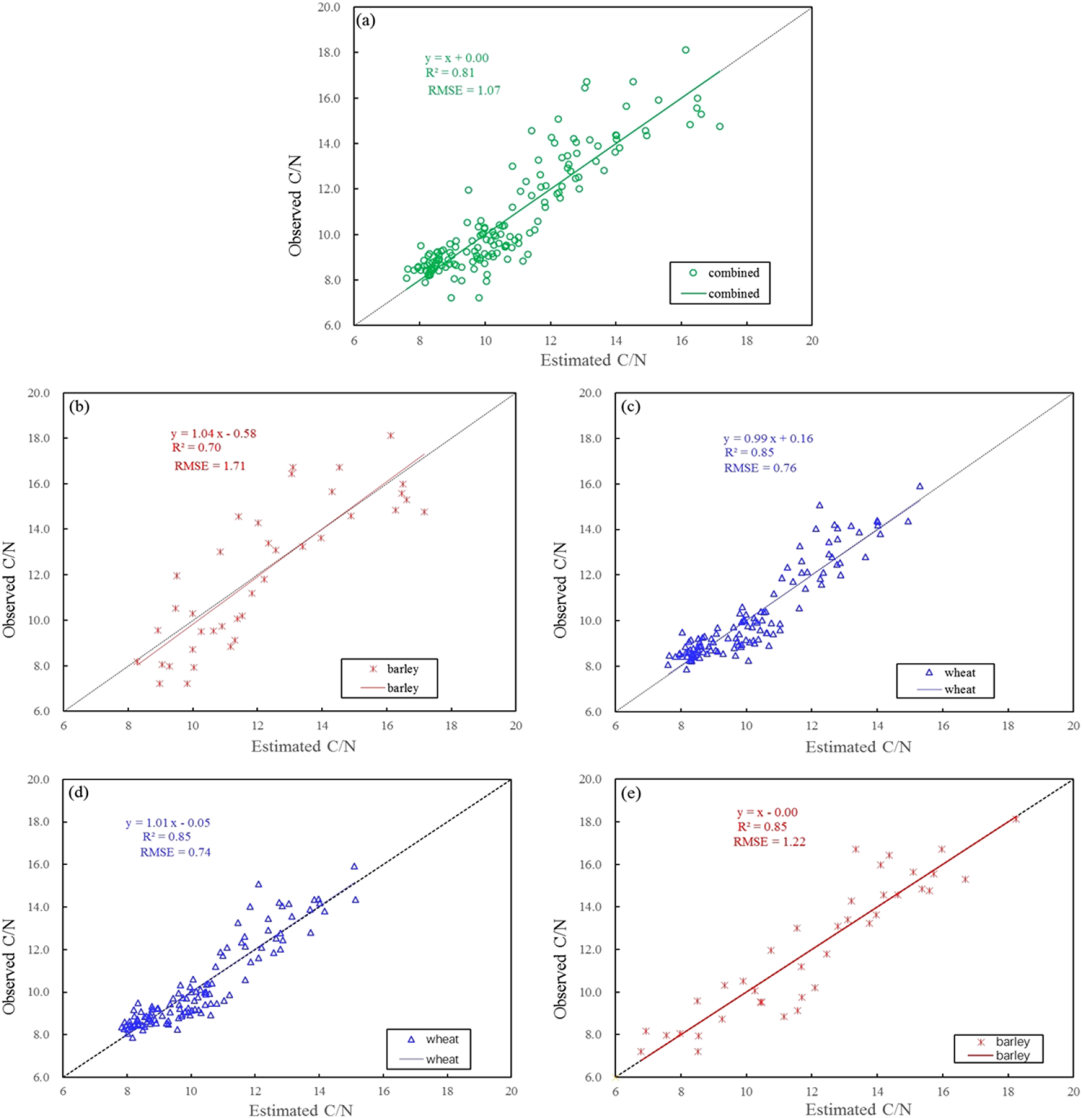
Relationships between the observed C/N and the estimated values using slope features: (**a**,**d** and **e**) denote the results of using the optimal slope feature models based on BB for combined, wheat and barley datasets, respectively, (**b** and **c**) indicate that of applying the model constructed by combined dataset to estimate C/N in wheat and barley, respectively.

Figure 11 displays the plotted relationships of the observed C/N to the estimated based on BB algorithm with slope features. Overall, the slopes of all linear fitting equations between the observed and estimated values are about 1, it explains that the estimations of leaf C/N is generally consistent with the observed, which indicates the BB method is very potential for estimating C/N in crops.

## Discussion

The study explored the feasibility of using slope features extracted from canopy hyperspectral reflectance curves to evaluate leaf C/N. In contrast with the existing spectral indices which have employed only a few wavelengths to monitor the biochemical constituents including N status, the slope characteristics make use of the abundant information from all bands on the segmented spectral curves and represent something of a refinement or simplification for all bands from the segmented spectral region. For instance, the slope features *K*_*pb*_, *K*_*ge*_ and *K*_*re*_ utilize the total bands within the ranges of 400–500 nm, 500–550 nm and 680–760 nm, respectively. From correlation analysis between leaf C/N and canopy reflectance in this study (Fig. 6), it can be found that most of the bands used by the above slope features are significantly related with leaf C/N except for the little range of 740–745 nm, which shows that these features from spectral curves are reasonable to some extent for leaf C/N estimates. On the other hand, as there exist high correlations between the neighbouring bands in hyperspectral reflectance data and canopy reflection are strongly influenced by soil background, view-sun-target geometry, canopy structure and properties that vary spatially and temporally^24–26,28,52^, spectral indices using only several wavebands to evaluate biochemical constituents might display the instability of the selected wavelengths when extrapolated to the other places and years^19^. Slope features presented in this study have synthetically utilized dozens even or hundreds of wavebands from the segmented reflectance curves, which helps to cope with the problem of high correlations between the neighbouring wavelengths in hyperspectral technology.

Although leaf C/N in crops has been influenced by the two factors of both LCC and LNC, the analyses from the present study illustrate that LCC varies gently, but LNC more strongly, so that leaf C/N as the ratio of LNC to LCC changes conspicuously with LNC, and properly speaking, significantly negatively correlated with LNC, but not notably with LCC. These conclusions also agree with the previous researches on leaf C/N from several forest vegetations, rice and corn^18,22,53^, and it implies that some methods of detecting N status with reflectance spectra may be applicable to leaf C/N. Although Curran^54^ and Fourty *et al*.^55^ found that the reflectance spectra based on dried and ground leaves were related to nitrogen and carbonaceous materials (such as cellulose, lignin and sugar) concentration with the characteristic absorption wavelengths in the 1400–2400 nm SWIR(short wave infrared) domain (for example, N absorption wavelengths are at 1510 nm, 19400 nm, 2060 nm, 2300 nm, etc.), those findings cannot be used for C/N estimates because reflectance spectra are commonly measured over fresh vegetation in fields rather than dry vegetation. For fresh green vegetation, these absorption features of N and carbonaceous materials are obscured by strong water absorptions centered at 1450 nm and 1940 nm^56^. So, in view of close linkage between N and chlorophyll in green leaves^29,38,39^, many N indicators have been proposed based on spectral characteristic of chlorophyll in visible and red-edge^57,58^. In this study, some existing indices considered to be good indicators of assessing N, chlorophyll and leaf C/N were tested to estimate Leaf C/N in wheat and barley, the two indices *MTCI* and *RI*_*1dB*_ that used the sensitive bands mainly from red-edge region showed the better performance of estimating leaf C/N for wheat, barley or combined dataset among the selected indices (Table 3), which implies that the reflectance spectra from red-edge region can be more useful for evaluating leaf C/N in two crops. The best feature (*K*_*ge*_ + *K*_*nir*_)/(2*K_*re*_) that is proposed to estimate leaf C/N also applies the information from red-edge region.

The results of correlation analysis between leaf C/N and slope features indicate that among the three typical slope features *K*_*pb*_, *K*_*ge*_, and *K*_*re*_ in visible region, only *K*_*re*_ extracted from red-edge region is the most negatively correlated with leaf C/N which means that the slope of a spectral curve within red-edge range will ascend with C/N decreasing, but for both *K*_*pb*_ and *K*_*ge*_ related positively to leaf C/N, the two slopes will ascend with C/N increasing. These cases agree well with changes of spectral reflectance curves from different C/N levels in Fig. 2a. Especially, the enlarged sketch describing slope features of spectral curves from Fig. 2b–e can better explain the responding relationships between C/N and slopes. Among the three slope features *K*_*nir*_, *K*_*nir2*_ and *K*_*nir3*_ in NIR region, the ones with more significant correlations can also describe well the dynamic pattern of C/N changes to some extent. In this study the analyses also show that the combined feature (*K*_*ge*_ + *K*_*nir*_)/(2*K_*re*_) has the most significantly positive correlation with leaf C/N in wheat and barley, which indicates that (*K*_*ge*_ + *K*_*nir*_)/(2*K_*re*_) decreases with C/N decreasing. Considered that N is the most demanding element for crop, C/N decreasing implies that high N concentration comes into being and chlorophyll concentration also increases due to close linkage between N and chlorophyll^29,38,39^. (*K*_*ge*_ + *K*_*nir*_)/(2*K_*re*_) uses spectral information from red and green regions where strong absorption and reflection are dominated by chlorophyll. When C/N decreases, the increase of chlorophyll concentration strengthens the absorption further for red light and it makes red well deeper. Accordingly, reflectance curves in red-edge region (680–760 nm) become steeper and their slopes rise, namely *K*_*re*_ increases. Simultaneously, spectral reflectance curves from high reflection platform (760–910 nm) in NIR become flat which will lead to the decrease of *K*_*nir*_. Besides, increasing chlorophyll also enhances the absorption for blue light, which makes spectral-curves flat in green reflection region (500–550 nm) and *K*_*ge*_ will decrease inevitably. Thus both (*K*_*ge*_ + *K*_*nir*_) decreasing and *K*_*re*_ increasing make the ratio (*K*_*ge*_ + *K*_*nir*_)/(2*K_*re*_) more sensitive to C/N with significantly positive correlation, and this can also explain why (*K*_*ge*_ + *K*_*nir*_)/(2*K_*re*_) has the best performance of estimating C/N. In addition, the analyses illustrate that the combined features have the better relationships with leaf C/N than single slope features, and this agrees well with the conclusion that the combined indices may show a better capacity of evaluating crop biochemical parameters due to weakening some effects of external factors such as soil background^58–60^. What’s more, in this study the combined features such as (*K*_*ge*_ + *K*_*nir*_)/(2*K_*re*_) display more significant correlations with leaf C/N not only for single crop data (wheat or barley) but also for the combined dataset while single slope features only show the close relationships with leaf C/N for wheat or barley (see Fig. 7a–c), it demonstrates that the combined features are more suitable for assessing C/N as potential species-independent indicators. At least, they should be appropriate spectral variables for wheat and barley.

This present study used slope features from spectral curves to preliminarily conduct the estimates for leaf C/N with an acceptable accuracy, but only a try for wheat and barley. Henceforth, it is very necessary to do more research on further understanding the interrelation between leaf C/N and slope features and developing the quantitative models that effectively and dynamically monitor leaf C/N for some specific growth stages of crops. Meantime, the studies on increasing crop types, cultivars and different environment areas to further examine the new spectral features will also be extremely needed.

## Conclusions

C/N that can mirror the metabolic status of carbon and nitrogen is a comprehensive indicator for evaluating the metabolic balance of carbon-nitrogen and diagnosing growth vigor in crop plants. Effectively monitoring the dynamic patterns of leaf C/N in crops helps to guide field managements and improve the ultimate formation of yield and quality in crop production. On the basis of the characteristics of the tall, low, broad or narrow shapes of canopy hyperspectral reflectance curves due to the differences in amounts or levels of biochemical compositions in crops, the present study proposed new spectral feature slopes to monitor leaf C/N in wheat and barley. It is proved that leaf C/N is closely related to LNC in two crops, and it is feasible and reliable to use hyperspectral reflectance measurements to monitor leaf C/N in crops. the combined feature (*K*_*ge*_ + *K*_*nir*_)/(2*K_*re*_) proposed newly in this study had the best performance for estimating leaf C/N by comparison with some existing indices and can be used to describe well the dynamic patterns of leaf C/N in wheat and barley. In addition, BB algorithm coupling with slope features may effectively improve the accuracy of leaf C/N estimations. It is concluded that using the spectral slope features extracted from canopy hyperspectral reflectance with BB method appears very promising and potential for remote monitoring leaf C/N in crops. The preliminary research of utilizing slope features can also provide new ideas and referential methods for hyperspectral monitoring of other biochemical constituents.

## References

[1] Hucklesby DP, Brown CM, Howell SE, Hageman RH (1971). Late spring applications of nitrogen for efficient utilization and enhanced production of grain and grain protein in wheat. Agron. J..

[2] Scheromm P, Martin G, Bergoin A, Autran. JC (1992). Influence of nitrogen fertilizer on the potential bread-baking quality of two wheat cultivars differing in their responses to increasing nitrogen supplies. Cereal Chem..

[3] Woodard HJ, Bly A (1998). Relationship of nitrogen management to winter wheat yield and grain protein in South Dakota. J. Plant Nutr..

[4] Guo, S., Dang, T. & Hao, D. Effects of fertilization on wheat yield, NO3–N accumulation and soil water content in semi-arid area of China. *Scientia Agri*. *Sin*. **38**(**4**), 754–760 (in Chinese with English abstract 2005).

[5] Jin, X., Xu, W., Zhang, L., Qin, F. & Huang, S. Influence of physiological characteristics at grain filling stage on dying green of wheat plants in different varieties. *Acta Agron*. *Sin*. **20**(**1**), 99–105 (in Chinese with English abstract 1994).

[6] Roth GW, Fox RH (1989). Plant tissue test for predicting nitrogen fertilizer requirement of winter wheat. Agron. J..

[7] Vos J, Bom M (1993). Handheld chlorophyll meter: a promising tool to assess the nitrogen status of potato foliage. Potato Res..

[8] Castelli F, Contillo R, Miceli F (1996). Non-destructive determination of leaf chlorophyll content in four crop species. J. Agron. Crop Sci..

[9] Johnkutty I, Mathew G, Thiyagarajan TM, Balasubramanian V (2000). Relationship among leaf nitrogen content, SPAD and LCC values in rice. J. Trop. Agric..

[10] Botha EJ, Leblon B, Zebarth B, Watmough J (2007). Non-destructive estimation of potato leaf chlorophyll from canopy hyperspectral reflectance using the inverted PROSAIL model. Int. J. Appl. Earth Obs..

[11] Feng W, Yao X, Zhu Y, Tian Y, Cao W (2008). Monitoring leaf nitrogen status with hyperspectral reflectance in wheat. Eur. J. Agron..

[12] Vane G, Goetz AFH (1993). Terrestrial imaging spectrometry: current status, future trends. Remote Sens. Environ..

[13] Sims DA, Gamon JA (2002). Relationships between leaf pigment content and spectral reflectance across a wide range of species, leaf structures and developmental stages. Remote Sens. Environ..

[14] Hansen PM, Schjoerring JK (2003). Reflectance measurement of canopy biomass and nitrogen status in wheat crops using normalized difference vegetation indices and partial least squares regression. Remote Sens. Environ..

[15] Pu R, Ge S, Kelly NM, Gong P (2003). Spectral absorption features as indicators of water status in coast live oak (*Quevcus agrifolia*) leaves. Int. J. Remote Sens..

[16] Wang, X. Huang, J., Li, Y. & Wang, R. Study on hyperspectral remote sensing estimation models for the ground fresh biomass of rice. *Acta Agron*. *Sin*. **29**, 815–821 (in Chinese with English abstract 2003).

[17] Zhang Y, Chen JM, Miller JR, Noland TL (2008). Leaf chlorophyll content retrieval from airborne hyperspectral remote sensing imagery. Remote Sens. Environ..

[18] Shi, R., Niu, Z. & Zhuang D. Feasibility of estimating leaf C/N ratio with hyperspectral remote sensing data. *Remote Sens*. *Technol*. *Appl*. **18**(**2**), 76–80 (in Chinese with English abstract 2003).

[19] Shi, R., Niu, Z. & Zhuang D. Research on the effects of leaf biochemical concentrations on leaf spectra: case study of inversion of C:N ratio based on the absorption features centered at 2100 nm. *J*. *Remote Sens*. **9**(**1**), 1–7 (in Chinese with English abstract 2005).

[20] Xue, L., Yang, L. & Fan, X. Estimation of nitrogen content and C/N in rice leaves and plant with canopy reflectance spectra. *Acta Agron*. *Sin*. **32**(**3**), 430–435 (in Chinese with English abstract 2006).

[21] Feng, W. *et al*. Monitoring the sugar to nitrogen ratio in wheat leaves with hyperspectral remote sensing. *Scientia Agri*. *Sin*. **41**(**6**), 1630–1639 (in Chinese with English abstract 2008).

[22] Zhou, D. *et al*. C/N content ratio of rice leaf monitoring based on canopy hyperspectral parameters. *Trans*. *CSAE*. **25**(**3**), 135–141 (in Chinese with English abstract 2009).

[23] Baret, F. Vegetation canopy reflectance, Factors of variation and application for agriculture. In: Hunt, D. (Eds), Physical measurements and signatures in remote sensing. Courchevel, France. pp. 145–167 (1991).

[24] Baret F, Guyot G (1991). Potentials and limits of vegetation indices for LAI and APAR assessment. Remote Sens. Environ..

[25] Gobron N, Pinty B, Verstraete MM (1997). Theoretical limits to the estimation of the leaf area index on the basis of visible and near-infrared remote sensing data. IEEE Trans. Geosci. Remote Sens..

[26] Zarco-Tejada PJ, Rueda CA, Ustin SL (2003). Water content estimation in vegetation with MODIS reflectance data and model inversion methods. Remote Sens. Environ..

[27] Haboudane D, Miller JR, Pattey E, Zarco-Tejada PJ, Strachan. IB (2004). Hyperspectral vegetation indices and novel algorithms for predicting green LAI of crop canopies: modeling and validation in the context of precision agriculture. Remote Sens. Environ..

[28] Houborg R, Anderson M, Daughtry C (2009). Utility of an image-based canopy reflectance modeling tool for remote estimation of LAI and leaf chlorophyll content at the field scale. Remote Sens. Environ..

[29] Yoder BJ, Pettigrew-Crosby RE (1995). Predicting nitrogen and chlorophyll content and concentrations from reflectance spectra (400–2500 nm) at leaf and canopy scales. Remote Sens. Environ..

[30] Gregory PAB (1998). and Biochemical Sources of Variability in Canopy Reflectance. Remote Sens. Environ..

[31] Nguyen HT, Lee B (2006). Assessment of rice leaf growth and nitrogen status by hyperspectral canopy reflectance and partial least square regression. Eur. J. Agron..

[32] Xu X (2011). Study on relationship between new characteristic parameters of spectral curve and chlorophyll content for rice. Spectrosc. Spectr. Anal..

[33] Jiang CZ, Ishihara K, Satoh K, Katoh S (1999). Loss of the photosynthetic capacity and protein in senescence leaves at positions of two cultivars of rice in relation to source capacity of the leaves for carbon and nitrogen. Plant Cell Physiol..

[34] Tingey DT (2003). Elevated CO2 and temperature alter nitrogen allocation in Douglas-fir. Global Change Biol..

[35] Xu X, Zhao C, Wang J, Li C, Yang X (2013). Associating new spectral features from visible and near infrared regions with optimal combination principle to monitor leaf nitrogen concentration in barley. J. Infrared Millim. W..

[36] Yu B, Ostland M, Gong P, Pu R (1999). Penalized linear discriminant analysis of *in situ* hyperspectral data for conifer species recognition. IEEE Trans. Geosci. Remote Sens..

[37] Pu R (2009). Broadleaf species recognition with *in situ* hyperspectral data. Int. J. Remote Sens..

[38] Daughtry CST, Walthall CL, Kim MS, Brown de Colstoun E, McMurtrey JE (2000). Estimating corn leaf chlorophyll concentration from leaf and canopy reflectance. Remote Sens. Environ..

[39] Botha EJ, Zebarth BJ, Leblon B (2006). Non-destructive estimation of potato leaf chlorophyll and protein contents from hyperspectral measurements using the PROSPECT radiative transfer model. Can. J. Plant Sci..

[40] Jongschaap EE, Booij R (2004). Spectral measurements at different spatial scales in potato: relating leaf, plant and canopy nitrogen status. Int. J. Remote Sens..

[41] Narendra PM, Fukunaga K (1977). A branch and bound algorithm for feature subset selection. IEEE Trans. Comput..

[42] Furnival GM, Wilson RW (1974). Regressions by Leaps and Bounds. Technometrics..

[43] Viña A, Gitelson AA (2005). New developments in the remote estimation of the fraction of absorbed photosynthetically active radiation in crops. Geophys. Res. Lett..

[44] Tarpley L, Reddy KR, Sassenrath-Cole GF (2000). Reflectance indices with precision and accuracy in predicting cotton leaf nitrogen concentration. Crop Sci..

[45] Simone G, Wilhelm C (2003). Quantifying nitrogen status of corn (Zea mays L.) in the field by reflectance measurements. Eur. J. Agron..

[46] Zhu Y (2006). Monitoring leaf nitrogen in wheat using canopy reflectance spectra. Can. J. Plant Sci..

[47] Horler DNH, Dockray M, Barber J (1983). The red edge of plant leaf reflectance. Int. J. Remote Sens..

[48] Horler DNH, Dockray M, Barber J, Barringer AR (1983). Red edge measurements for remotely sensing plant chlorophyll content. Adv. Space Res..

[49] Miller JR, Hare EW, Wu J (1990). Quantitative characterization of the vegetation red edge reflectance model. Int. J. Remote Sens..

[50] Cho MA, Skidmore AK (2006). A new technique for extracting the red edge position from hyperspectral data: the linearextrapolation method. Remote Sens. Environ..

[51] Gong P, Pu R, Heald RC (2002). Analysis of *in situ* hyperspectral data for nutrient estimation of giant sequoia. Int. J. Remote Sens..

[52] Houborg R, Soegaard H, Boegh E (2007). Combining vegetation index and model inversion methods for the extraction of key vegetation biophysical parameters using Terra and Aqua MODIS reflectance data. Remote Sens. Environ..

[53] Jin X, Yang G, Tan C, Zhao C (2015). Effects of nitrogen stress on the photosynthetic CO_2_ assimilation, chlorophyll fluorescence, and sugar-nitrogen ratio in corn. Sci. Rep..

[54] Curran PJ (1989). Remote sensing of foliar chemistry. Remote Sens. Environ..

[55] Fourty T, Baret F, Jacquemoud S, Schmuck G, Verdebout J (1996). Leaf optical properties with explicit description of its biochemical composition: direct and inverse problems. Remote Sens. Environ..

[56] Kokaly RF, Clark RN (1999). Spectroscopic determination of leaf biochemistry using band-depth analysis of absorption features and stepwise multiple linear regression. Remote Sens. Environ..

[57] Reyniers M, Walvoort DJJ, De Baardemaaker J (2006). A linear model to predict with a multi-spectral radiometer the amount of nitrogen in winter wheat. Int. J. Remote Sens..

[58] Chen P (2010). New spectral indicator assessing the efficiency of crop nitrogen treatment in corn and wheat. Remote Sens. Environ..

[59] Eitel JUH, Long DS, Gessler PE, Smith AMS (2007). Using *in*-*situ* measurements to evaluate the new RapidEye™ satellite series for prediction of wheat nitrogen status. Int. J. Remote Sens..

[60] Haboudane D, Miller JR, Tremblay N, Zarco-Tejada PJ, Dextraze L (2002). Integrated narrow-band vegetation indices for prediction of crop chlorophyll content for application to precision agriculture. Remote Sens. Environ..

[61] Vogelmann JE, Rock BN, Moss DM (1993). Red edge spectral measurements from sugar maple leaves. Int. J. Remote Sens..

[62] Gitelson AA, Gritz Y, Merzlyak MN (2003). Relationships between leaf chlorophyll content and spectral reflectance and algorithms for non-destructive chlorophyll assessment in higher plant leaves. J. Plant Physiol..

[63] Xue LH, Cao WX, Luo WH, Dai TB, Zhu Y (2004). Monitoring leaf nitrogen status in rice with canopy spectral reflectance. Agron. J..

[64] Gupta RK, Vijayan D, Prasad TS (2003). Comparative analysis of red edge hyperspectral indices. Adv. Space Res..

[65] Wang, X., Huang, J., Li, Y. & Wang, R. Correlation between chemical contents of leaves and characteristic variables of hyperspectra on rice field. *Trans*. *CSAE* **19**(**2**), 144–148 (in Chinese with English abstract 2003).

[66] Wu C, Niu Z, Tang Q, Huang W (2008). Estimating chlorophyll content from hyperspectral vegetation indices: Modeling and validation. Agr. Forest Meteorol..

[67] Peñuelas J, Gamon JA, Fredeen AL, Merino J, Field CB (1994). Reflectance indices associated with physiological changes in nitrogen- and water-limited sunflower leaves. Remote Sens. Environ..

[68] Datt B (1999). A new reflectance index for remote sensing of chlorophyll content in higher plants: tests using Eucalyptus leaves. J. Plant Physiol..

[69] Gitelson AA, Viña A, Ciganda V, Rundquist DC, Arkebauer TJ (2005). Remote estimation of canopy chlorophyll content in crops. Geophys. Res. Lett..

[70] Haboudane D, Tremblay N, Miller JR, Vigneault P (2008). Remote estimation of crop chlorophyll content using spectral indices derived from hyperspectral data. IEEE Trans. Geosci. Remote Sens..

[71] Rouse, J. W., Haas, R. H., Schell, J. A., Deering, D. W. & Harlan, J. C. Monitoring the vernal advancements and retrogradation (green wave effect) of natural vegetation. In: NASA/GSFC, Type III, Final Report, Greenbelt, MD, USA, 1–371 (1974).

[72] Dash J, Curran PJ (2004). The MERIS terrestrial chlorophyll index. Int. J. Remote Sens..

[73] Fitzgerald GJ (2006). Spectral and thermal sensing for nitrogen and water status in rainfedand irrigated wheat environments. Precis. Agric..

[74] Feng W (2016). Remote detection of canopy leaf nitrogen concentration in winter wheat by using water resistance vegetation indices from *in*-*situ* hyperspectral data. Field Crops Res..

